# The evolutionary diversity of locomotor innovation in rodents is not linked to proximal limb morphology

**DOI:** 10.1038/s41598-019-57144-w

**Published:** 2020-01-20

**Authors:** Brandon P. Hedrick, Blake V. Dickson, Elizabeth R. Dumont, Stephanie E. Pierce

**Affiliations:** 1 Department of Cell Biology and Anatomy, School of Medicine, Louisiana State University Health Sciences Center, New Orleans, LA 70112 USA; 2 Museum of Comparative Zoology and Department of Organismic and Evolutionary Biology, Harvard University, Cambridge, MA 02138 USA; 3 Department of Earth Sciences, University of Oxford, Oxford, UK; 4 School of Natural Sciences, University of California–Merced, Merced, CA 95343 USA

**Keywords:** Evolution, Palaeontology, Speciation, Ecology

## Abstract

Rodents are the most species-rich order within Mammalia and have evolved disparate morphologies to accommodate numerous locomotor niches, providing an excellent opportunity to understand how locomotor innovation can drive speciation. To evaluate the connection between the evolutionary success of rodents and the diversity of rodent locomotor ecologies, we used a large dataset of proximal limb CT scans from across Myomorpha and Geomyoidea to examine internal and external limb shape. Only fossorial rodents displayed a major reworking of their proximal limbs in either internal or external morphology, with other locomotor modes plotting within a generalist morphospace. Fossorial rodents were also the only locomotor mode to consistently show increased rates of humerus/femur morphological evolution. We propose that these rodent clades were successful at spreading into ecological niches due to high behavioral plasticity and small body sizes, allowing them to modify their locomotor mode without requiring major changes to their proximal limb morphology.

## Introduction

Major evolutionary innovations are often followed by a burst of taxonomic diversification and a period of rapid morphological change as species adapt to new ecological niches^1–8^. While major evolutionary radiations can often be traced back to the origin of a completely novel structure (e.g., wings in birds, bats, and pterosaurs), smaller-scale radiations have also been tied to increased morphological adaptability. For example, morphological variation in the skulls of stenodermatine bats have allowed specialization on fruit rather than insects, which in turn led to an increase in their speciation rates (e.g.,^9–14^). While the linkage between morphological novelties and evolvability, taxonomic diversification, and the invasion of novel niches is often hypothesized, it is rarely tested quantitatively because this requires both repeated convergence and a well-resolved phylogeny for estimating diversification. As the most specious mammalian order with exceptional taxonomic and ecological diversity, Rodentia meets both these criteria and thus provides an ideal study group for understanding the relationship between adaptation and diversification^15,16^.

Rodents are native to every continent except Antarctica^17^ and biogeographical diversification has been considered a likely driver behind extant rodent diversity^15,16,18,19^. However, more recent studies have demonstrated that most colonizations of new continents by rodents (with the exception of sigmodontine rodents in South America^20^) have not been followed by increases in rates of speciation^18,19^, suggesting that factors beyond biogeographical expansion, such as morphological innovation, may have led to the present rodent diversity. Although the unique feeding apparatus of rodents is often associated with their evolutionary success^21–23^, rodents also encompass a wide variety of locomotor ecologies that are characterized by morphological adaptations. For example, ricochetal rodents such as jerboas and kangaroo rats have long hindlimbs relative to their forelimbs^24^ while fossorial species such as blind mole rats have expanded forelimbs suited to their digging lifestyle^25^. Further, traditional morphometrics of rodent appendicular skeletons has been successful at differentiating locomotor modes^26,27^. Considering the apparent link between limb morphology and ecology, it can be hypothesized that rodents deviating from a terrestrial, generalist locomotor niche, may also significantly deviate in limb shape to better accommodate their locomotor requirements. In turn, limb adaptation may have affected rates of morphological evolution and disparity, thus influencing speciation rates as rodents filled previously unavailable niches.

**Figure 1:**
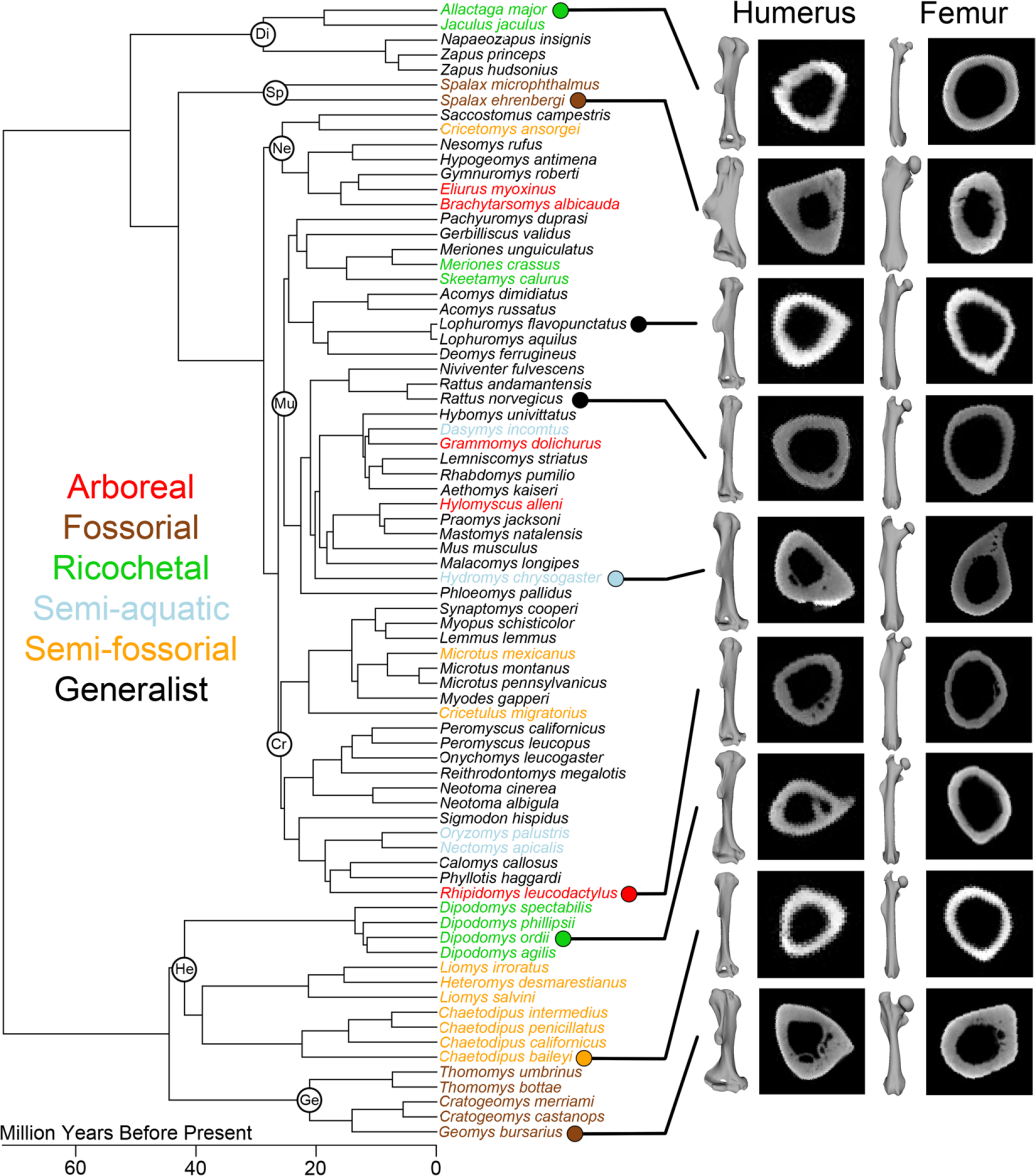
Phylogeny of all taxa included showing primary locomotory mode by color. Note multiple examples of convergence for each category across the tree. Representative cross-sections and three-dimensional models of humeri and femora from taxa from each locomotor category are shown on the right with lollipops indicating to which taxon they belong. Larger clades are identified by circles: Di = Dipodoidea, Sp = Spalacidae, Ne = Nesomyidae, Mu = Muridae, Cr = Cricetidae, He = Heteromyidae, Ge = Geomyidae.

Here, we utilized the remarkable diversity of myomorph and geomyoid rodents as a basis to robustly test how locomotor ecology affects limb morphology and rates of morphological evolution. We set out to answer two major questions: **(1)** What is the relationship between locomotor ecology and the external and internal morphology of proximal limb bones in these rodent clades? and **(2)** Do shifts in locomotor ecology correlate with bursts of morphological evolution? Further, how do rate shifts in morphological evolution compare with previously published shifts in rodent speciation rates?^15,16^. To accomplish this, we analyzed the humeri and femora of 76 species from 203 individual specimens of rodents from across the Myomorpha and the Geomyoidea representing six locomotor ecologies (generalist, arboreal, fossorial, ricochetal, semi-fossorial, and semi-aquatic) using micro-computed tomography (µCT) and measured external and internal bone shape (see methods; Fig. 1; Table S1). External shape was quantified using a three-dimensional pseudolandmark geometric morphometric (3DGM) approach^28^ and two internal cross-sectional geometry (CSG) parameters were measured that represent bone strength in compression, bending, and torsion^29,30^. These data were then used to examine morphological evolution of the proximal limb across the rodent phylogeny using maximum likelihood estimates and Bayesian analysis^31,32^.

## Results

### Internal and external limb shape and ecology (Question 1)

**Figure 2:**
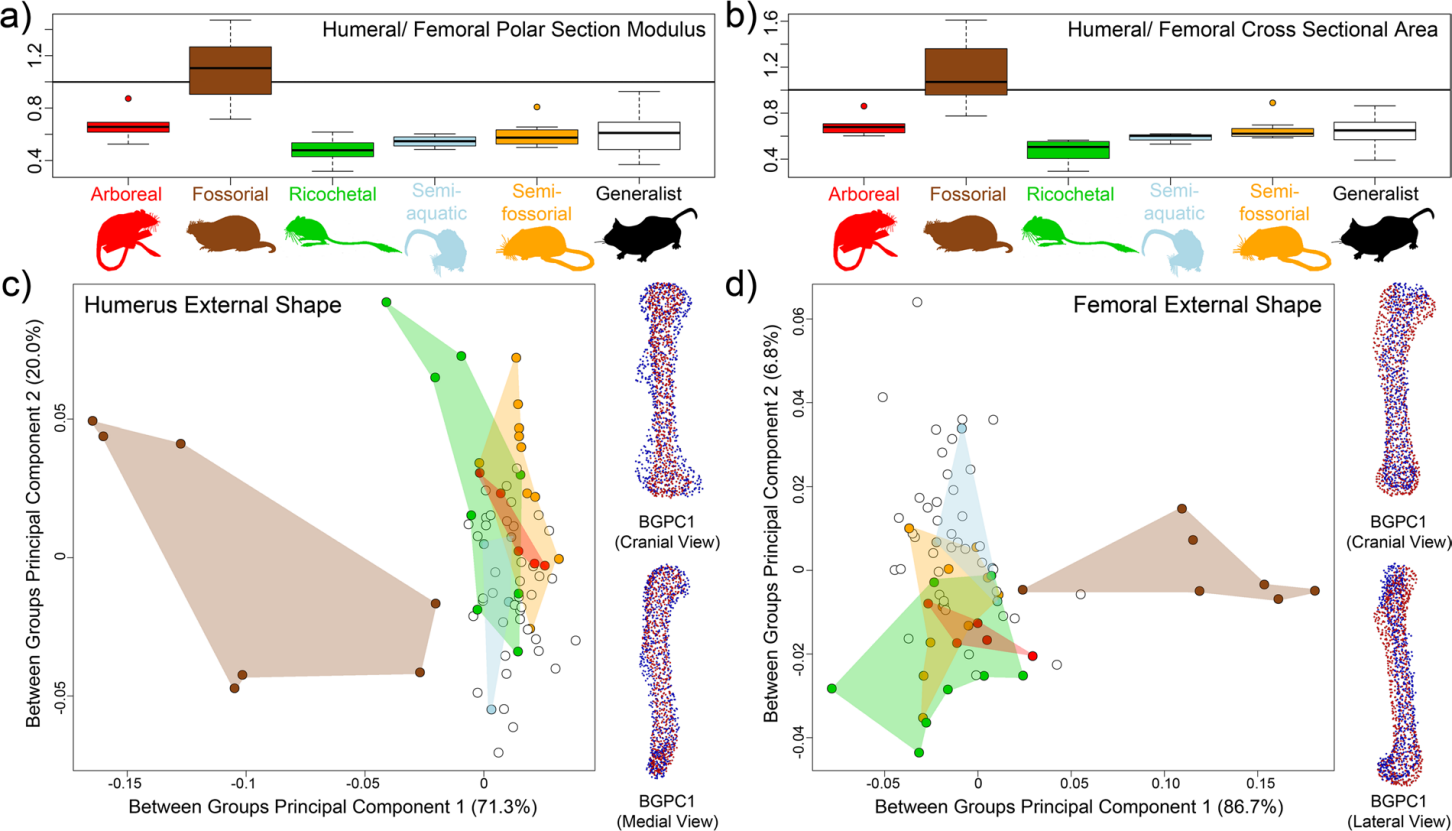
Differences in cross-sectional geometry and external bone shape by locomotor mode. Boxplots showing on the x-axis (**a**) the relative humerus:femur polar section modulus (Zpol) and (**b**) humerus:femur cross-sectional area (CSA). Values greater than one indicate a relatively strong humerus relative to femur whereas lower values indicate stronger femora relative to humeri. Between-groups PCAs for (**c**) humerus external shape and (**d**) femur external shape with non-generalist ecological modes colored by hulls. White dots represent generalist taxa and are not surrounded by a hull. To the right of each bgPCA are example humeri and femora showing the extremes of bgPC1. The humerus is shown in cranial and medial view and the femur in cranial and lateral views. Red points represent the positive end of the bgPC1 axis and blue points represent the negative end.

Two internal geometric properties were measured at the midshaft: the polar section modulus (a measure of bending and torsional strength) and cross-sectional area (compressional strength), following previous studies^29,30^. Both the relative humerus-femur polar section modulus (Zpol) and relative cross-sectional area (CSA) showed significant differences between locomotor ecological categories using phylogenetic corrections (Table 1). However, this was driven solely by fossorial taxa and no groups were significantly different from one another except for fossorial taxa (Fig. 2a,b). Fossorial rodents were significantly different from ricochetal and semi-fossorial rodents in their Zpol and from all ecologies other than arboreal rodents in CSA (Tables 1, S2). The lack of separation between arboreal rodents and fossorial rodents was likely a result of the small sample size characterizing arboreal rodents (n = 5). Although not significantly different from groups other than fossorial rodents, ricochetal rodents had stronger femora relative to their humeri in comparison with other groups (Fig. 2a,b).

**Table 1:** Internal bone cross-sectional parameters showing phylogenetically corrected pairwise p-values for different ecologies with comparisons in the polar section modulus above the diagonal and cross-sectional area below the diagonal. A = arboreal, F = fossorial, R = ricochetal, Sa = semi-aquatic, Sf = semi-fossorial, and G = generalist. The t-values for the pairwise comparisons can be found in Table S2.

	A	F	R	Sa	Sf	G
A		0.22	1	1	1	1
F	0.132		**0**.**015**	0.065	**0**.**015**	0.072
R	1	**0**.**015**		1	1	1
Sa	1	**0**.**039**	1		1	1
Sf	1	**0**.**015**	1	1		1
G	1	**0**.**048**	1	1	1

Between-groups principal component analyses (bgPCA)^33^ of 3D landmark coordinates (see methods) of both humerus and femur external shape separated fossorial rodents from all other locomotor ecologies with other locomotor ecologies strongly overlapping (Fig. 2c,d). For the humerus, bgPC1 summarized 71.3% of total variation. Taxa with highly positive bgPC1 values (red landmark configuration) had slender humeri with a reduced deltopectoral crest as compared to taxa with highly negative bgPC1 values (blue landmark configuration), which had robust distal condyles and a pronounced deltopectoral crest (Fig. 2c). In contrast, bgPC2 summarized 20% of total variance and did not show any noticeable patterns. When examining humerus shape using Procrustes shape data against ecology with a Procrustes MANOVA with pairwise comparisons, fossorial taxa were significantly different from all other locomotor ecologies, but no other ecologies were significantly different from one another (Tables 2, S3).

**Table 2:** Pairwise comparisons incorporating phylogeny for external limb shape for the humerus (above the diagonal) and femur (below the diagonal). A = arboreal, F = fossorial, R = ricochetal, Sa = semi-aquatic, Sf = semi-fossorial, and G = generalist. The effect sizes for the pairwise comparisons can be found in Table S3.

	A	F	R	Sa	Sf	G
A		**0**.**001**	0.729	0.98	0.676	0.769
F	**0**.**001**		**0**.**001**	**0**.**002**	**0**.**001**	**0**.**001**
R	0.816	**0**.**001**		0.917	0.706	0.82
Sa	0.935	**0**.**001**	0.731		0.671	0.979
Sf	0.841	**0**.**001**	0.791	0.976		0.409
G	0.341	**0**.**001**	0.428	0.979	0.983	

The femur bgPC1 summarized 86.7% of total variation and also separated fossorial rodents from all other locomotor ecologies with other ecologies strongly overlapping. Taxa with highly positive bgPC1 values (red landmark configuration) had robust, straight femora with large greater trochanters and distal condyles (Fig. 2d). Taxa with highly negative bgPC1 values (blue landmark configuration) had slender, slightly sigmoidal femora (Fig. 2d). Some ricochetal rodents plotted on the negative axis of bgPC2 with generalist rodents tending towards the positive axis. However, bgPC2 only accounted for 6.8% of total variation and thus the difference was not substantial. As with the humerus, Procrustes MANOVAs revealed significant differences between femur shape (Procrustes shape data) of fossorial taxa and all other locomotor ecologies. No other ecological groupings were significantly different from one another (Tables 2, S3).

Both humerus and femur external shape (Procrustes shape data) were significantly correlated with phylogeny (K_mult_ = 0.4096, p < 0.001; K_mult_ = 0.373, p < 0.001, respectively). Humerus shape, and not femur shape, had a significant, but weak allometric trend (R.^2^ = 0.043, p = 0.004; R.^2^ = 0.019, p = 0.149, respectively) (Table S4). However, the allometric trend for humerus shape was primarily driven by fossorial taxa. These taxa were not only some of the largest specimens in terms of centroid size, but also had the most exaggerated shapes. The removal of fossorial taxa eliminated the significant allometric trends. Because of our interest in locomotor modes including fossoriality, the likelihood that the shape of fossorial elements was related to digging and not size, and the low percent of total variation explained by allometry, we did not use allometrically corrected residuals.

**Figure 3:**
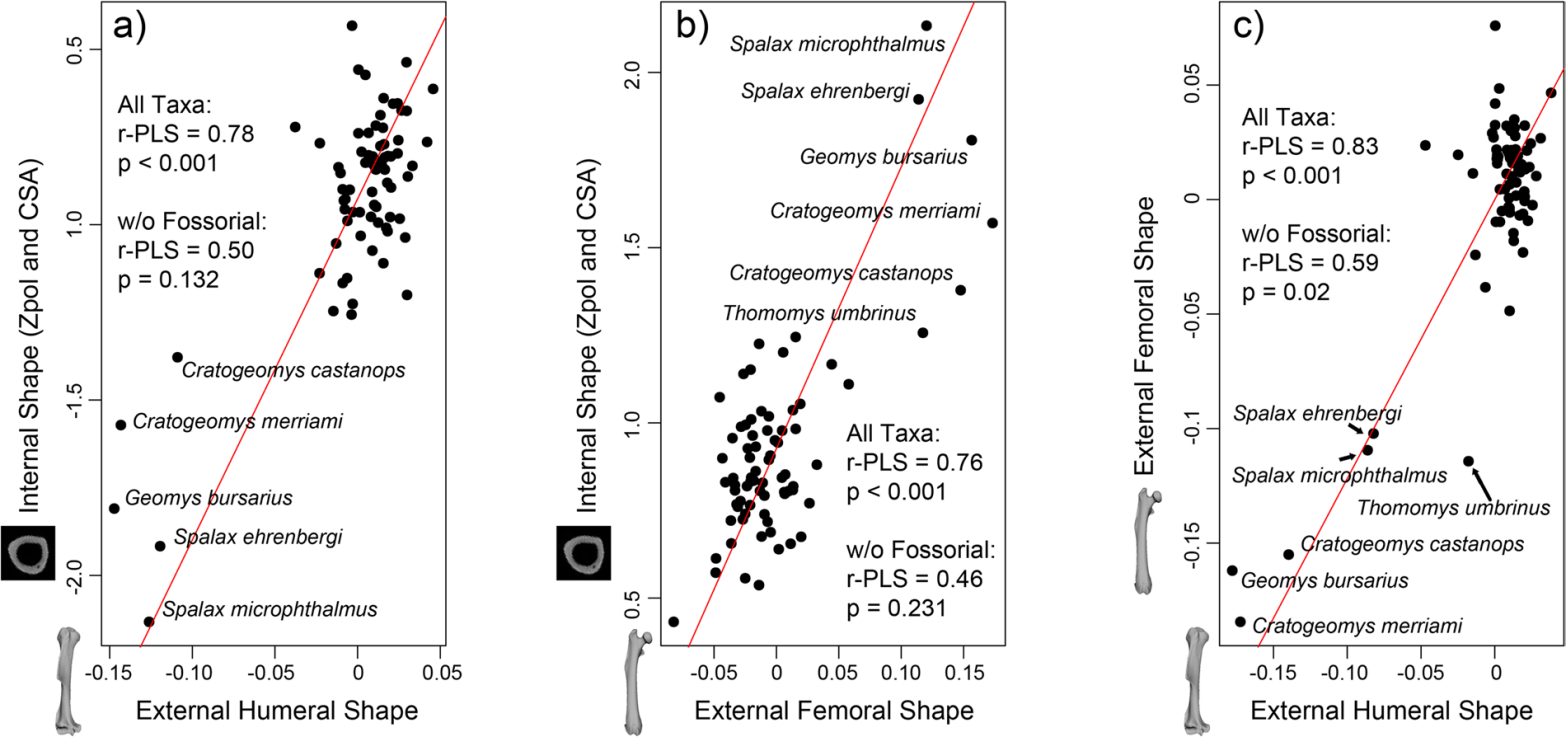
Partial least squares analyses of (**a**) combined humerus and femur internal shape by external humerus shape, (**b)** internal shape by external femur shape, and (**c**) external humerus shape by external femur shape. Fossorial taxa are labelled and plot outside of the remaining taxa; the relationship between internal and external shape is no longer significant when fossorial taxa are removed from the analyses.

Comparing internal (relative humerus-femur Zpol and CSA) and external humerus shape (Procrustes shape data) using partial least squares (PLS) regressions revealed a significant association with locomotor ecology (r-PLS = 0.78, p < 0.001; Fig. 3a; Table S5). However, this trend was largely driven by the highly modified fossorial taxa and when they were removed from the analysis, the correlation was no longer significant (r-PLS = 0.50, p = 0.132). The association between internal limb geometry and external femur shape revealed a similarly significant correlation when fossorial taxa were included (r-PLS = 0.76, p < 0.001), but not when they were removed (r-PLS = 0.46, p = 0.231; Fig. 3b; Table S5). Further, external humerus shape and external femur shape were significantly correlated (r-PLS = 0.83, p < 0.001; Fig. 3c; Table S5). In this case, removing fossorial taxa did not eliminate the significant correlation (r-PLS = 0.59, p = 0.02).

### Ancestral states and shifts in morphological evolution (Question 2)

To examine morphological evolution of the proximal limb, ancestral state reconstructions were generated through maximum likelihood estimates for relative humerus-femur Zpol and CSA and for bgPC1 and bgPC2 for external humerus and femur shape. Further, shifts in the rate of morphological evolution were examined for relative humerus-femur Zpol, PC1 of humerus external shape (25% of total variation, from a standard principal components analysis, see Fig. S1), and PC1 of femur external shape (33.2% of total variation, Fig. S1) in a Bayesian context using Bayesian Analysis of Macroevolutionary Mixtures (BAMM)^31,32^. Rate shifts for external shape were also examined in a non-Bayesian likelihood-based multivariate context for comparison^34^.

**Figure 4:**
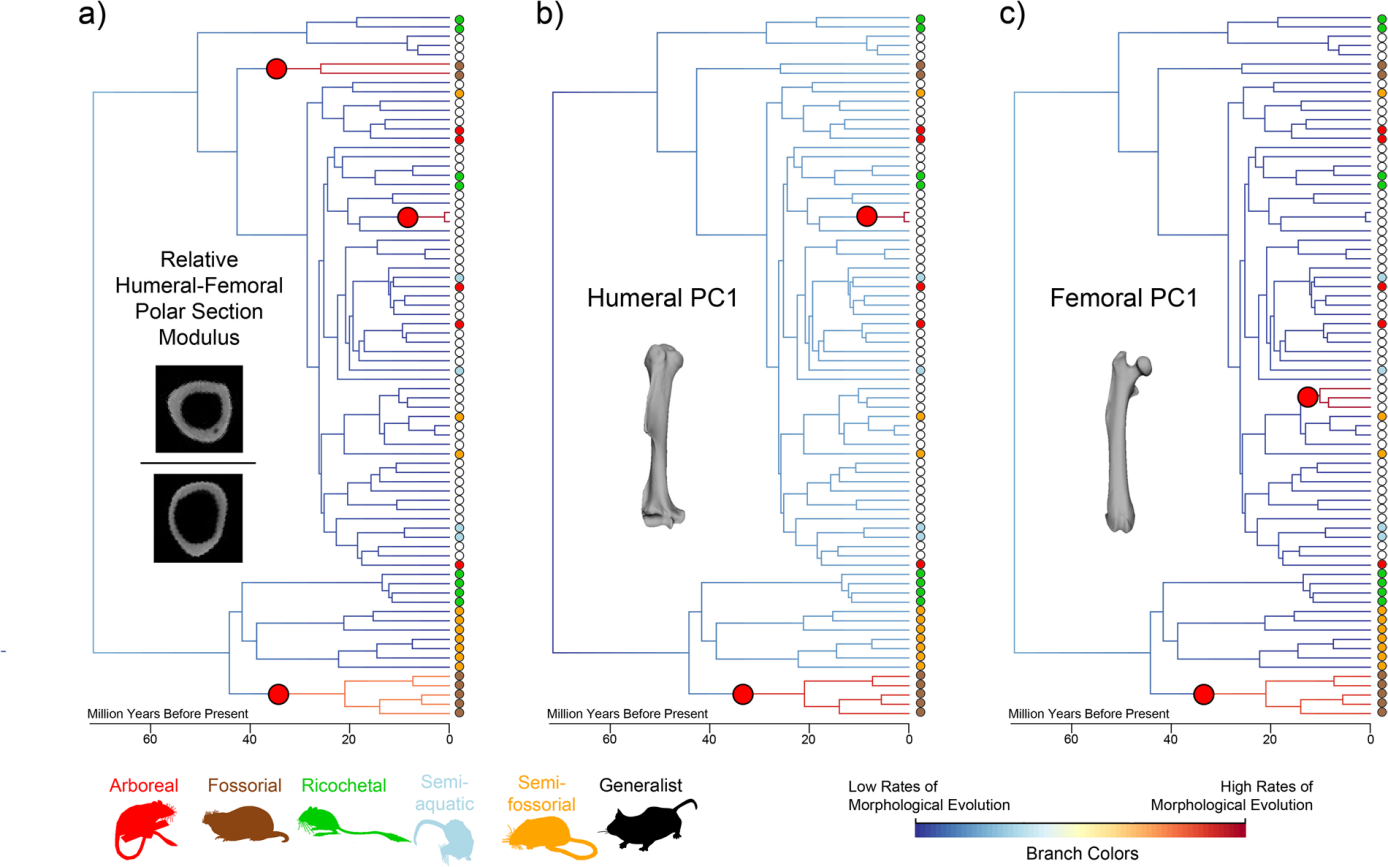
Shifts in rates of morphological evolution for (**a**) the relative humerus:femur polar section modulus, (**b**) PC1 of the humerus external shape, and (**c**) PC1 of the femur external shape. Tips are colored based on locomotor mode and significant shifts are represented by large red circles. Significant shifts were determined using a marginal odds ratio of the posterior probability of observing a shift and the prior probabilities of a branch having a shift due to branch length. These shifts represent the best shifts that were sampled most frequently under a BAMM framework. Branch colors show relatively high rates of morphological evolution (warmer colors) and relatively low rates of morphological evolution (cooler colors).

Maximum likelihood-based ancestral state reconstruction of internal geometry indicates that intermediate values for the relative humerus-femur Zpol were typical among taxa although the femur was often slightly stronger, with exceptionally strong humeri or femora evolving near tips, such as in *Geomys bursarius* (Fig. S2). From the root, the majority of clades (with the exception of fossorial clades) evolved femora that are stronger relative to their humeri, with jerboas (*Jaculus*, *Allactaga*) developing the strongest femora relative to their humeri. Maximum likelihood ancestral state reconstructions of relative humerus-femur CSA demonstrated similar trends with *Spalax* (mole rats) evolving exceptionally high humerus-femur CSA and jerboas evolving low humerus-femur CSA. Only three clades showed significant shifts in the rate of morphological evolution for the relative humerus-femur Zpol using BAMM: the fossorial *Spalax* spp. and geomyids, and the terrestrial *Lophuromys* spp. (brush-furred mice) (Figs. 4a, S3). These rate shifts generally mirror those found using likelihood-based methods with strong shifts occurring in fossorial taxa. However, ancestral state reconstructions also showed large differences between ricochetal taxa and generalists, a pattern not supported by BAMM.

Maximum likelihood ancestral state reconstruction of external geometry recovered that the vast majority of taxa have similar values for the humerus and femur, only developing noticeable trends on lineages leading to fossorial or ricochetal forms. The humerus bgPC1 ancestral state reconstruction demonstrated intermediate and low values on branches leading to geomyids, *Spalax* sp., and the ricochetal heteromyids (kangaroo rats and allies), but not along lineages to other heteromyids relative to the ancestral averages at the root of the tree (Fig. S4). Ricochetal heteromyids similarly have very high values for bgPC2, deviating from other heteromyids, which maintain values near the ancestral average. In the femur, bgPC1 shows geomyids and *Spalax* spp. having particularly high values along their lineages relative to the ancestral average, whereas other taxa developed low values relative to the root (Fig. S5).

Using a Bayesian-framework, two clades showed significant shifts in the rate of morphological evolution in external humerus PC1 shape, a clade containing geomyids and a clade containing *Lophuromys* spp. (Fig. 4b). These shifts mirror those found for internal geometry (Fig. 4a), with the exception of the exclusion of *Spalax* spp. Although it was not considered robust, ricochetal heteromyids had a change in morphological rate shift dynamics, evolving in the opposite direction from fossorial taxa (Fig. S6). Some iterations demonstrated significant shifts at the base of the heteromyid clade, but these were not considered robust (marginal odds ratios < 5). For external femur PC1 shape, geomyids once again showed a significant shift in the rate of morphological evolution relative to other clades (Fig. S7). Additionally, femur shape within the generalist clade composed of *Synaptomys cooperi*, *Myopus schisticolor*, and *Lemmus lemmus* (lemmings) also exhibited a significant shift in its rate of morphological evolution (Fig. 4c). Rate shifts analyzed using a likelihood-based multivariate approach using the first 25 PC axes showed the same two strongly supported shifts for external humerus shape found in the BAMM analysis (fossorial geomyids and *Lophuromys* sp.), but also found a shift in the fossorial *Spalax* spp. (Fig. S8a). In contrast, a likelihood-based multivariate analysis on femur shape (first 25 PC axes) did not align with the results of the BAMM analysis (Fig. S8b). All shifts occurred in terminal generalist taxa and not in clades.

The only clade recovered with significant shifts in morphological rate dynamics across all three Bayesian analyses (relative humerus-femur polar section modulus, external humerus shape, external femur shape) were geomyids and the only non-generalist locomotor group with significant shifts were fossorial rodents.

## Discussion

Despite the large niche space afforded to myomorph and geomyoid rodents by their high locomotor diversity, only fossorial taxa have significantly different proximal limb shapes when compared to other ecological groups (generalist, arboreal, ricochetal, semi-fossorial, and semi-aquatic). This was true of both internal limb geometry (measured using cross-sectional geometry (CSG) parameters) and external limb shape (measured using 3DGM) (Fig. 2). The proximal limb elements of myomorph and geomyoid rodents adapted to locomotor ecologies other than fossoriality were indistinguishable from those of generalist taxa, indicating that most myomorph and geomyoid rodents share a generalized proximal limb form. Additionally, fossorial clades are the only non-generalist rodents to consistently recover increased rates of evolution in internal or external proximal limb bone shape (Fig. 4). Our result is in keeping with Alhajeri *et al*.^19^ who found that basic morphological measurements (e.g., body length, tail length, etc.) were not correlated with increases in muroid rodent disparity–leading to the idea that phylogenetic diversity and morphological disparity may be decoupled in rodents, as has been found in other groups (e.g.,^35^).

Myomorph and geomyoid rodents also do not undergo allometrically-driven appendicular modifications to their proximal limbs (Fig. S9). Although humerus shape was significantly correlated with allometry, this association was driven entirely by fossorial taxa. Sansalone *et al*.^36^ recently found a similar allometric relationship in the humeri of moles (contra prior works^37,38^), suggesting that underground environments may impose strong selective pressures on both the size and shape of the humerus whereby fossoriality may require a larger body size than other ecologies^36^. For example, the fossorial rodent with the smallest mean femur length in our dataset (*Spalax ehrenbergi*) is larger than 50% of the rodents in our sample. Previous studies have suggested that rodents in general have minimal allometric trends with quadrupedal and bipedal rodent limbs having the same scaling relationships^39^ such that the smallest birch mouse has the same forelimb-hindlimb ratio as the largest rat^40^. Even within humeri and femora, the cortical area and the second moment of inertia scale isometrically^41^, meaning that as bone length increases, cortical bone is not more periosteally distributed^42^. It is possible that non-fossorial rodents within our sample do not possess allometric increases in external bone shape as they encompass a rather small body size range, and all have a ‘crouched’ limb posture with similar biomechanical demands^43,44^. Small mammals are likely overbuilt for the loads that they typically experience, and only large mammals require major allometry-driven morphological changes^45^. This relaxed allometric constraint may be one of the factors allowing rodents to adapt to new ecological niches without the need to significantly alter their proximal limb morphology.

Our data indicate that proximal limb bone internal geometry and external shape vary independently for the majority of rodent ecologies. Combined forelimb and hindlimb internal geometry (Zpol_hum_/Zpol_fem_, CSA_hum_/CSA_fem_ standardized by element length) is not significantly associated with either humerus or femur external shape for myomorphs and geomyoids with non-fossorial locomotor modes (Fig. 3a,b). This is supported by Dawson^46^, who suggested that due to their small size, small mammals may not follow Wolff’s law (i.e. when a bone is loaded over time, it will remodel in response to that loading). However, internal and external geometry are correlated when including the extreme morphologies found in fossorial rodent taxa. Previous studies have also found fossorial rodents to have CSG parameters that are significantly different from terrestrial generalists. Biknevicius *et al*.^47^ found that CSG parameters were successful at distinguishing burrowing rodents (*Ctenomys*) from non-burrowing rodents to the point that the rigidity of the humerus of *Ctenomys* exceeded that of brachiating primates^48^. Interestingly, internal and external geometry may be decoupled in fossorial moles^37^, suggesting that non-rodents may achieve fossoriality via different morphological pathways^38^. In the past, numerous workers have used either internal or external bone shape to assess locomotor mode in a wide variety of clades (e.g.,^29,30,42,49–56^), including rodents^41,47^. However, internal and external proximal limb bone shape data appear to have high morphological plasticity within rodents and do not necessarily evolve together. So, although there are clear trends within fossorial taxa, more work needs to be done on the connection between internal and external shape data for inferring locomotion in non-fossorial rodents.

Further, shifts in the rates of proximal limb bone evolution do not appear to align with shifts in speciation rates within the Myomorpha and Geomyoidea^15,16^. Within the subset of rodent clades we examined, Fabre *et al*.^16^ found 20 significant shifts in diversification rates, one in the Castorimorpha (Castoridae and Geomyoidea), none within the Dipodidae, and 19 within Muroidea. The shift found within Castorimorpha by Fabre *et al*.^16^ occurred within the fossorial Geomyidae and was not related to the significant increase in rates of morphological evolution that we found at the base of fossorial geomyids (Fig. 4). Within muroids, none of the shifts in speciation rates were associated with any locomotor grouping based on our study^15,16^. Venditti *et al*.^57^ similarly found no sustained shifts in rates of body size evolution in Rodentia. These results do not mean that morphology did not play a role in the diversification of myomorph and geomyoid rodents into new niches, only that the shape of the proximal limb was not a primary driver. Other factors, such as the evolution of the elongate distal limb of ricochetal rodents^24^ or webbed feet and insulatory fur in semi-aquatic rodents like the Australian water rat (*Hydromys chrysogaster*)^58^ may provide better reflections of ecology. This is perhaps best seen in holistic studies of the appendicular skeleton using linear rather than geometric morphometrics^26,27^, which have successfully separated rodent locomotor modes. However, these studies were not performed using phylogenetic comparative methods. Given the significant association between proximal limb shape and phylogeny in our dataset, this is likely a confounding variable and needs to be included in future studies.

Based on our results, we propose that diversity in myomorph and geomyoid rodent locomotor ecology is linked to their generalist morphology and high behavioral plasticity rather than the evolution of novel proximal limb morphologies^59^. The diversity of locomotory ecologies, and their repeated evolution, is probably facilitated by the small body size range of the rodents studied, relaxing both morphological and biomechanical constraints and reducing selective pressure for structural modification of the proximal limb bones^37,46^. Additional factors, such as environmental change^60–62^ may also have led to increased taxonomic diversification in rodents. Muroids, in particular, have been hugely successful at range expansions and novel introductions to new regions in comparison with more morphologically specialized groups or those with multiple locomotor modes^14^. These results in modern rodents allude to how small, generalist early mammals may have been so successful at quickly proliferating into multiple locomotor modes following niche openings after the Cretaceous-Tertiary boundary^63,64^.

## Materials and Methods

### Sample, data collection, and ecological categories

The sample included rodent humeri and femora from n = 76 species (k = 203 specimens) ranging across seven families within the Myomorpha and their sister group, the Geomyoidea (Cricetidae, n = 20; Muridae, n = 26, Dipodidae, n = 5, Nesomyidae, n = 7, Spalacidae, n = 2, Heteromyidae, n = 11, Geomyidae, n = 5). These clades were chosen because their constituent taxa have multiple convergences in locomotory mode. Where possible, multiple individuals per species were sampled to account for intraspecific variability (Fig. S10). All specimens were wild caught, expect for the lesser Egyptian jerboa (*Jaculus jaculus*), which were captive bred. All specimens were accessioned in museum collections at the time of this study, none were killed as part of the study, and all necessary permissions from museums were obtained prior to study. Sex was not specified when selecting specimens due to limitations in museum collections and previous studies showing little sexual dimorphism in muroids^19^.

To quantify both internal and external shape, rodent humeri and femora were scanned in batches using a Nikon Metrology HMXST225 μCT system (Nikon Metrology, Inc., Tokyo, Japan) at the Center for Nanoscale Systems at Harvard University. Scan parameters were optimized based on the size and thickness of the bones in each scan, but were generally performed at 70 kV and 70 μA with a resolution of 40 μm. Image stacks were generated using the proprietary CTPro software (Nikon Metrology Inc., Tokyo, Japan) and cropped so that each bone was represented by a single stack. Finally, image stacks were segmented in Mimics v. 16.0 (Materialise, Leuven, Belgium) and 3D models were smoothed, made watertight, and exported in STL format in 3Matic (Materialise, Leuven, Belgium).

Each species was assigned to one of six commonly used locomotor categories^26,65–67^ based on an exhaustive review of the literature: arboreal (n = 5), generalist (n = 42), ricochetal (n = 8), semi-fossorial (n = 10), fossorial (n = 7), and semi-aquatic (n = 4) (Table S1). Definitions for these groups follow Samuels and Van Valkenburgh^26^. Specifically, semi-aquatic taxa regularly swim to avoid predators or obtain food, arboreal taxa spend substantial time climbing to avoid predators or obtain food, semi-fossorial taxa spend substantial time constructing simple burrows, fossorial taxa build complex underground burrows and predominantly live underground in those burrows, and ricochetal rodents locomote primarily through bipedal saltation^26^. Generalist rodents may perform some of these behaviors, but do not do so extensively. We do note that these commonly used rodent locomotor categories are a mixture of functional categories (e.g., fossorial) and habitat categories (e.g., arboreal). More work on classifying rodent behaviors (and mammalian behaviors more generally) would undoubtedly improve our classifications.

### Phylogeny

To account for the influence of phylogeny on our data^68^, a species-level, time-calibrated phylogenetic tree was created by pruning the phylogeny of Fabre *et al*.^16^ in the R package *APE*^69^ to incorporate only the species included in this study. As in previous studies^13,70^, species means were taken for shape parameters (below) such that each phylogenetic tip was represented by a single value for each parameter.

### Cross-sectional bone geometry

To evaluate cross-sectional parameters, image stacks were imported into ImageJ^71^ and the BoneJ plug-in^42,72^ was used to calculate the polar section modulus (Zpol) and cross-sectional area (CSA) of each bone. Zpol combines bending strengths in all planes and torsional strength simultaneously, thus representing overall bone strength; CSA measures the strength of the bone under compression where larger CSA values confer greater resistance to compression. Zpol considers both bending and torsional strength whereas the second moment of area (I_max_/I_min_) only considers bending strength and thus the second moment of area was not used. In addition to these parameters, relative humerus-femur strength was assessed by calculating the ratio of Zpol_hum_ (normalized by humerus length) to Zpol_fem_ (normalized by femur length), and relative humerus-femur CSA by calculating the ratio of CSA_hum_ to CSA_fem_^30^. Cross-sectional parameters were measured at 40% midshaft length for the humeri to accommodate the distal position of the deltopectoral crest and at 50% midshaft length for the femora.

To check for correlations between the humerus internal shape parameters and femur internal shape parameters, Phylogenetic Generalized Least Squares (PGLS) analyses were run in the package *nlme*^73^. Restricted maximum likelihood was used to simultaneously estimate the value of Pagel’s lambda (where 0 is equivalent to a star phylogeny and 1 is equivalent to Brownian motion) and a regression line was fit to compare Zpol_hum_ and Zpol_fem_ and CSA_hum_ and CSA_fem_ for the humeri and femora separately. To assess differences between locomotor categories amongst these parameters, phylogenetic ANOVAs and pairwise comparisons were run on the correlation between locomotor categories and relative humerus-femur Zpol and CSA respectively in the package *phytools*^74^. These comparisons were graphically represented using boxplots.

### External bone shape

To quantify external bone shape, an automatic landmarking method developed by Boyer *et al*.^28,75^ and written for R as *auto3dgm* was utilized. Long bone shape can be particularly difficult to quantify comprehensively using regular geometric morphometric methods due to a dearth of anatomically homologous landmarks when analyzing a wide range of taxa with divergent long bone shapes. Therefore, normal landmarking techniques would leave a large proportion of the bone shape unsampled. Instead, this method applies geometrically homologous pseudolandmarks to the 3D surface mesh algorithmically such that landmarks are applied serially to areas that are least sampled. It is important to note that the pseudolandmarks are geometrically and not biologically homologous, but this is not conceptually different from other techniques such as Fourier analysis, eigenshape analysis, or eigensurface analysis^28^. The landmark configurations are then iteratively adjusted to minimize pairwise Euclidian distances among specimens. Initial alignment of the data was performed using 250 landmarks and then refined using 768 landmarks.

Following alignment of shapes, landmark data were imported into R through the *geomorph* package^76^ and aligned using Procrustes superimposition. Data were then examined using both traditional principal component analyses (PCA) and between-groups principal components analysis (bgPCA) with ecological groups as the grouping variables^33^ in *Morpho*^77^ (Tables S6–9). bgPCA is similar to PCA, but projects data onto orthogonal principal components of group averages (Tables S7–S9). Although a recent publication has suggested caution when applying bgPCA^78^, this analysis was only used to represent data graphically (bgPCA plots and mapping ancestral shape data onto the phylogeny) and all statistical analyses were performed on shape data (Procrustes aligned coordinates), not on bgPCs. The impact of allometry on the data was then estimated by regressing the common allometric component (CAC) on the log mean of bone length for both the humerus and femur external shape datasets^79^. Note that bone length measurements are not ideal for allometric studies, but the vast majority of specimens included in our analyses did not have body mass data recorded. The strength of phylogenetic signal in the data was assessed using the multivariate multiple K-statistic with significance determined through 999 permutations^80^. Finally, external shape of the humerus and femur were compared between ecological groups using a phylogenetically informed Procrustes ANOVA with pairwise comparisons.

### Internal versus external bone shape

To compare internal and external shape data, a two-block partial least squares (PLS) analysis^81^ was used with internal and external shape in separate blocks^82^. Three separate analyses were conducted: (1) comparing humerus external shape to internal parameters, (2) comparing femur external shape to internal parameters, and (3) comparing humerus external shape to femur external shape. Internal parameters included both the relative humerus-femur Zpol and relative humerus-femur CSA. Significance was determined using 999 permutations. Given a substantial difference between fossorial taxa and other ecological categories, these analyses were run once with all taxa and again with fossorial taxa removed.

### Ancestral state reconstruction and rates of morphological evolution

To examine how external and internal bone shape evolved across the rodent phylogeny, maximum likelihood ancestral state reconstructions were generated for four parameters: relative humerus-femur Zpol, relative humerus-femur CSA, and bgPC1 and bgPC2 shape variables for both the humerus and femur. Values were mapped onto the phylogeny as a heatmap, where larger values were cool and smaller values were hot. Further, Bayesian Analysis of Macroevolutionary Mixtures (BAMM) was used to calculate rates of morphological evolution for the relative humerus-femur Zpol, PC1, and PC2 within a Bayesian context^31,32^. Prior to calculating shifts, reasonable priors were found based on the tree’s branch lengths using *BAMMtools* (Table S10). BAMM was then run in terminal (Mac OS10.9.5) and convergence of the generated MCMC was tested using *coda*^83^. Whereas other methods for calculating rates of morphological evolution rely on AICs, BAMM simulates a posterior distribution of shift probabilities on a phylogenetic tree. These marginal shift probabilities are the probability that a shift occurred on a specific branch. To account for the fact that longer branches will be more likely to have shifts than shorter branches, a ratio (marginal odds ratio) of the posterior probability of observing a shift and the prior probabilities of a branch having a shift due to branch length was calculated. Credible rate shifts with marginal odds ratios greater than five were then calculated and these data were condensed into a macroevolutionary cohort matrix visually showing how different lineages share, or do not share, common rate shift dynamics.

Additionally, a second analysis of rates of morphological evolution was run using MOTMOT^34^, an approach that models trait evolution in a non-Bayesian multivariate context. These analyses were run on humerus and femur external shape data using the first 25 axes of a principal component analysis, which summarized greater than 90% of total shape variation in both datasets. MOTMOT analyses were run using the ‘tm1’ model, which does not specify the location of any shifts *a priori*, and using 4 total shifts.

## Supplementary information

Supporting Information.

Supporting Information2
