# Supplementary material for: The evolutionary diversity of locomotor innovation in rodents is not linked to proximal limb morphology
Source: Sci Rep. 2020 Jan 20;10:717. doi: 10.1038/s41598-019-57144-w (PMC6970985; doi:10.1038/s41598-019-57144-w)
Supplement: Supplementary file 1 — Supporting Information. [file 41598_2019_57144_MOESM1_ESM.pdf]

The evolutionary diversity of locomotor innovation in rodents is not linked to proximal limb morphology

Brandon P Hedrick[1,2,3,*], Blake V Dickson[2], Elizabeth R Dumont[4], Stephanie E Pierce[2,*]

[1]Department of Cell Biology and Anatomy, School of Medicine, Louisiana State University Health Sciences Center, New Orleans, LA 70112, USA; [2]Museum of Comparative Zoology and Department of Organismic and Evolutionary Biology, Harvard University, Cambridge, MA 02138, USA; [3]Department of Earth Sciences, University of Oxford, Oxford UK; [4]School of Natural Sciences, University of California–Merced, Merced, CA 95343, USA

*for correspondence: bphedrick1@gmail.com (BPH), spierce@oeb.harvard.edu (SEP)

Competing interests: The authors declare no competing interests

The evolutionary diversity of locomotor innovation in rodents is not linked to proximal limb morphology

Supplementary files

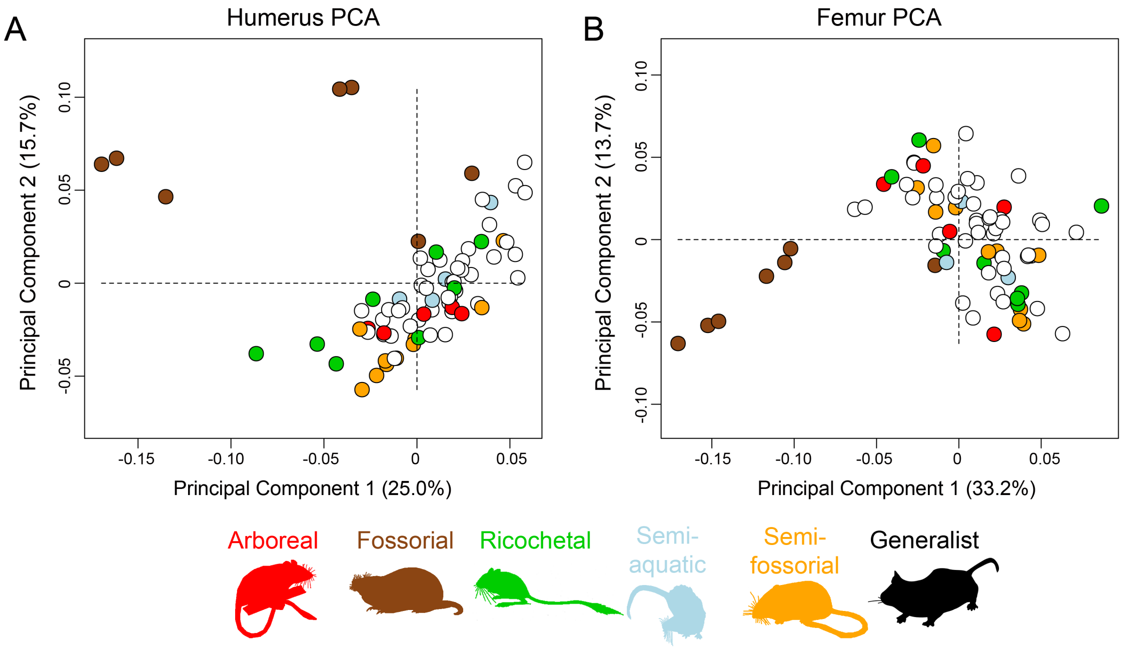

Figure S1: Principal component analysis of the (**a**) humerus and (**b**) femur with ecological groups colored.

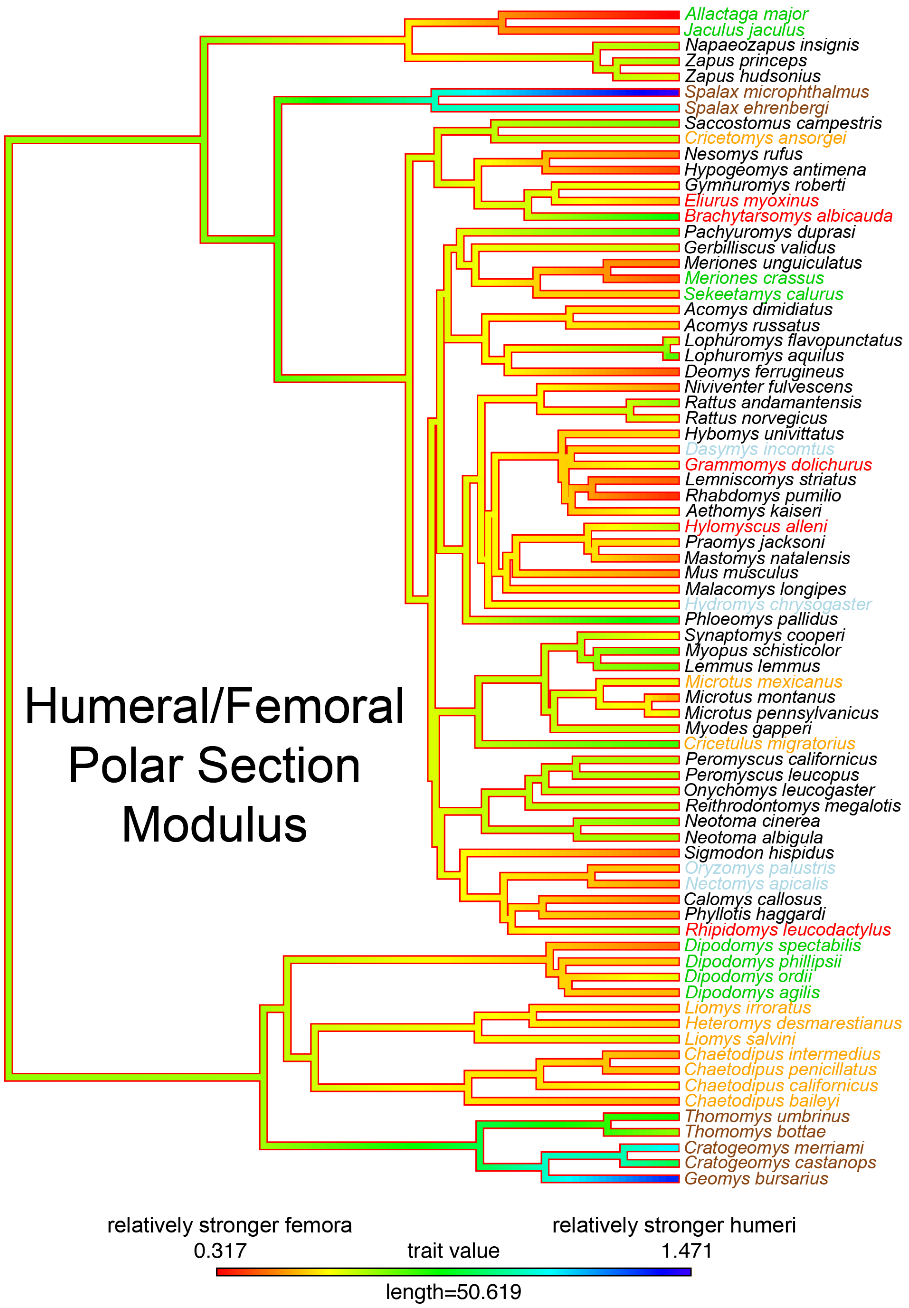

Figure S2: Heat map showing changes in relative humerus-femur polar section modulus values across the phylogeny. Hotter colors represent lower values (weaker humerus relative to femur) and colder colors represent higher values (stronger humerus relative to femur).

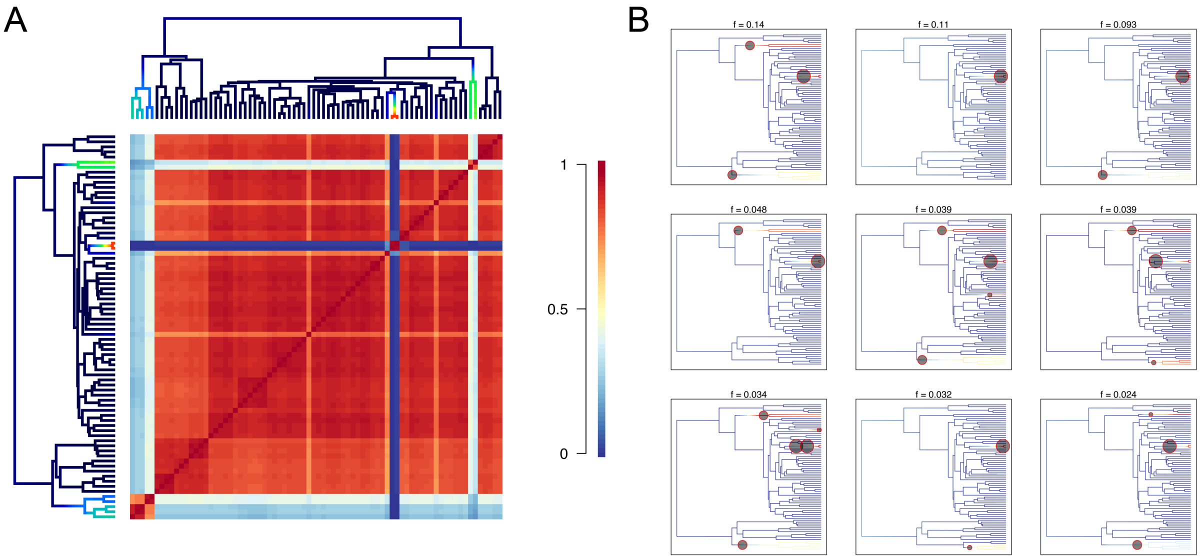

Figure S3: (**a**) Macroevolutionary cohort matrix showing the relative strength of the BAMM shifts for the relative humerus-femur polar section modulus. Hotter colors indicate taxa evolving along the same macroevolutionary regime and colder colors indicate taxa evolving at different macroevolutionary regimes. (**b**) Set of credible shifts using a marginal prior to posterior odds ratio threshold of 5 (figure 3 indicates only the “best shift”).

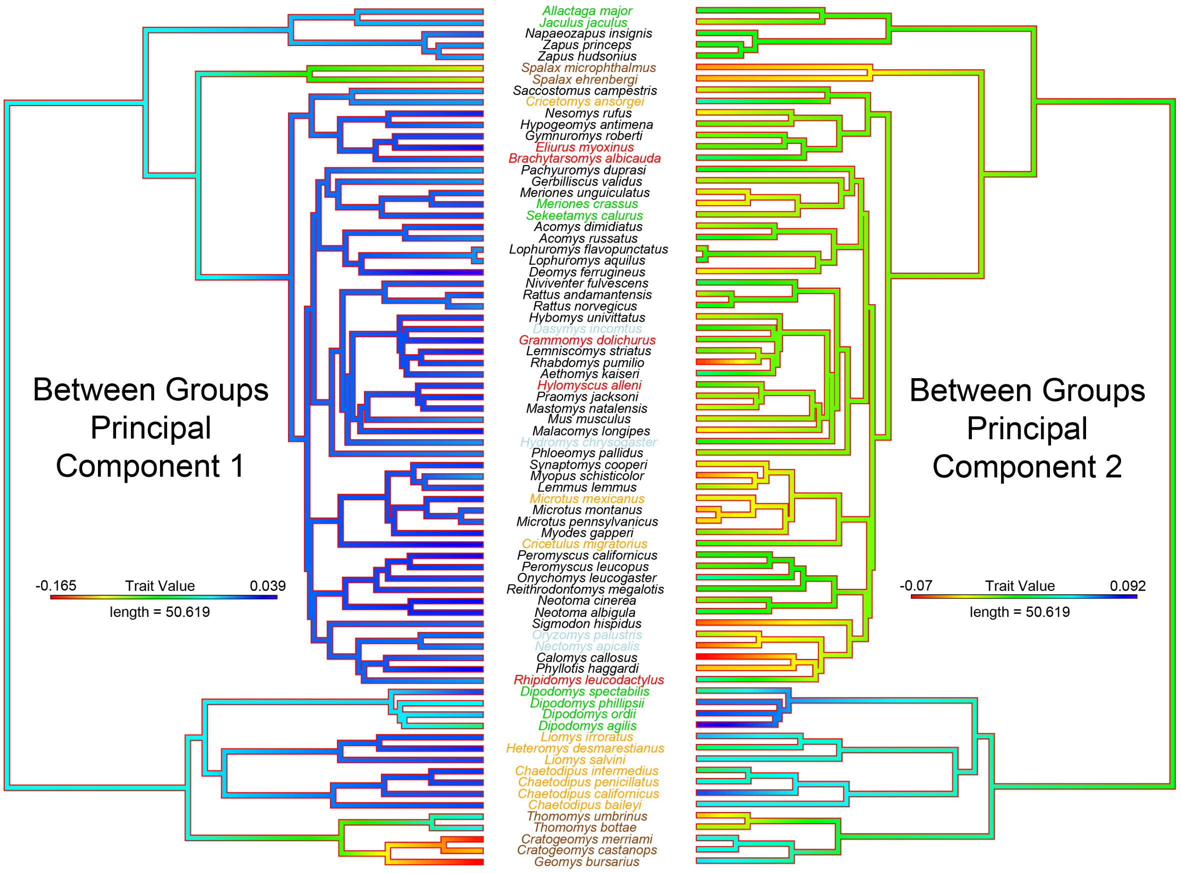

Figure S4: Heat map showing changes in between-groups principal component 1 (**left**) and between-groups principal component 2 (**right**) of the external humerus shape across the phylogeny. Hotter colors represent lower bgPC values and colder colors represent higher bgPC values. Note that a combination of bgPC1 and bgPC2 distinguish fossorial taxa and low bgPC2 values separate heteromyids (both semi-fossorial and ricochetal forms).

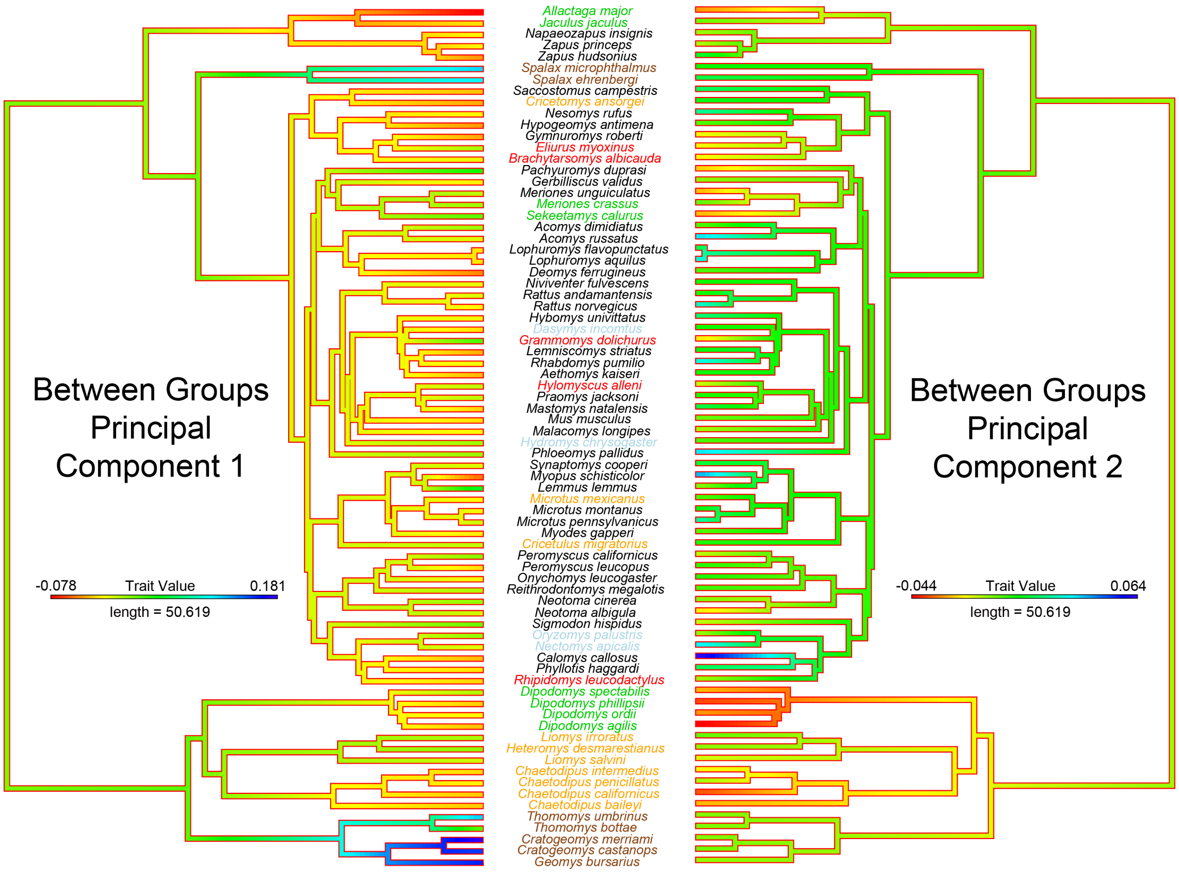

Figure S5: Heat map showing changes in between-groups principal component 1 (**left**) and between-groups principal component 2 (**right**) of the external femur shape across the phylogeny. Hotter colors represent lower bgPC values and colder colors represent higher bgPC values.

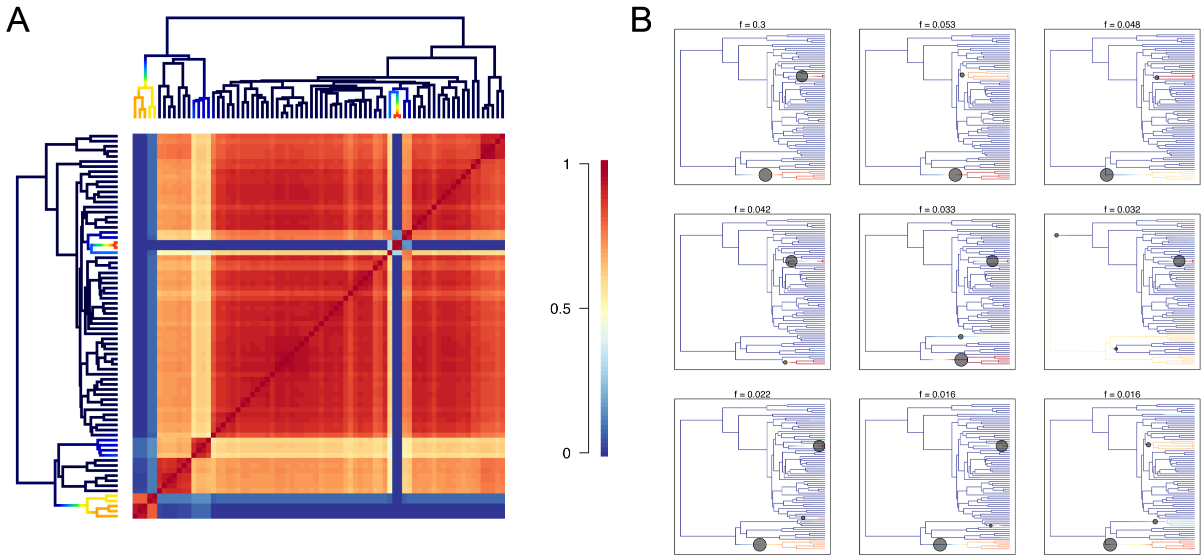

Figure S6: (**a**) Macroevolutionary cohort matrix showing the relative strength of the BAMM shifts for principal component 1 of the external humerus shape. Hotter colors indicate taxa evolving along the same macroevolutionary regime and colder colors indicate taxa evolving at different macroevolutionary regimes. (**b**) Set of credible shifts using a marginal prior to posterior odds ratio threshold of 5 (figure 3 indicates only the “best shift”).

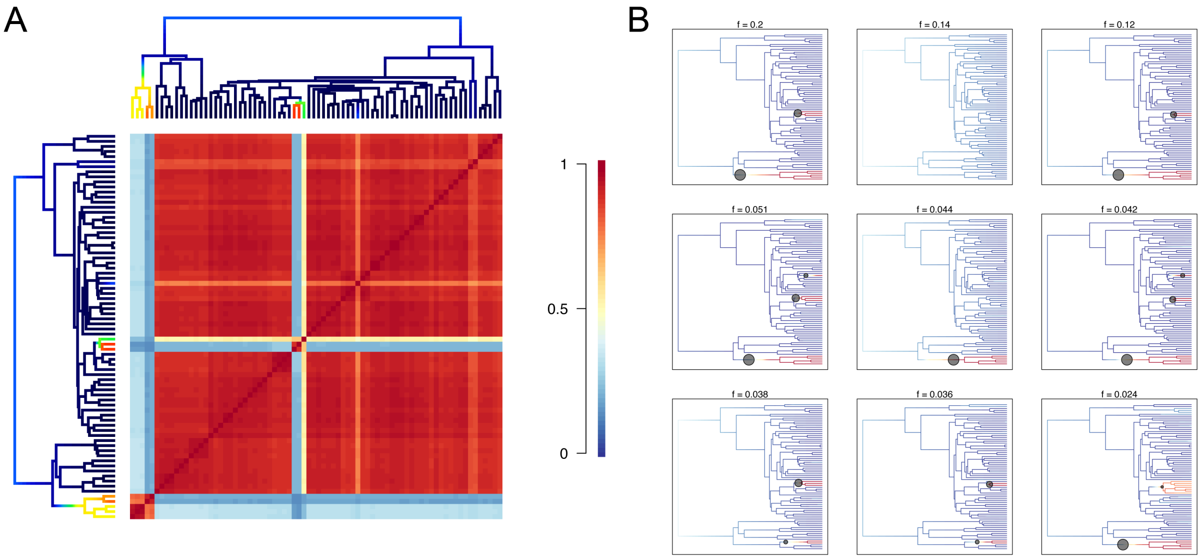

Figure S7: (**a**) Macroevolutionary cohort matrix showing the relative strength of the BAMM shifts for principal component 1 of the external femur shape. Hotter colors indicate taxa evolving along the same macroevolutionary regime and colder colors indicate taxa evolving at different macroevolutionary regimes. (**b**) Set of credible shifts using a marginal prior to posterior odds ratio threshold of 5 (figure 3 indicates only the “best shift”).

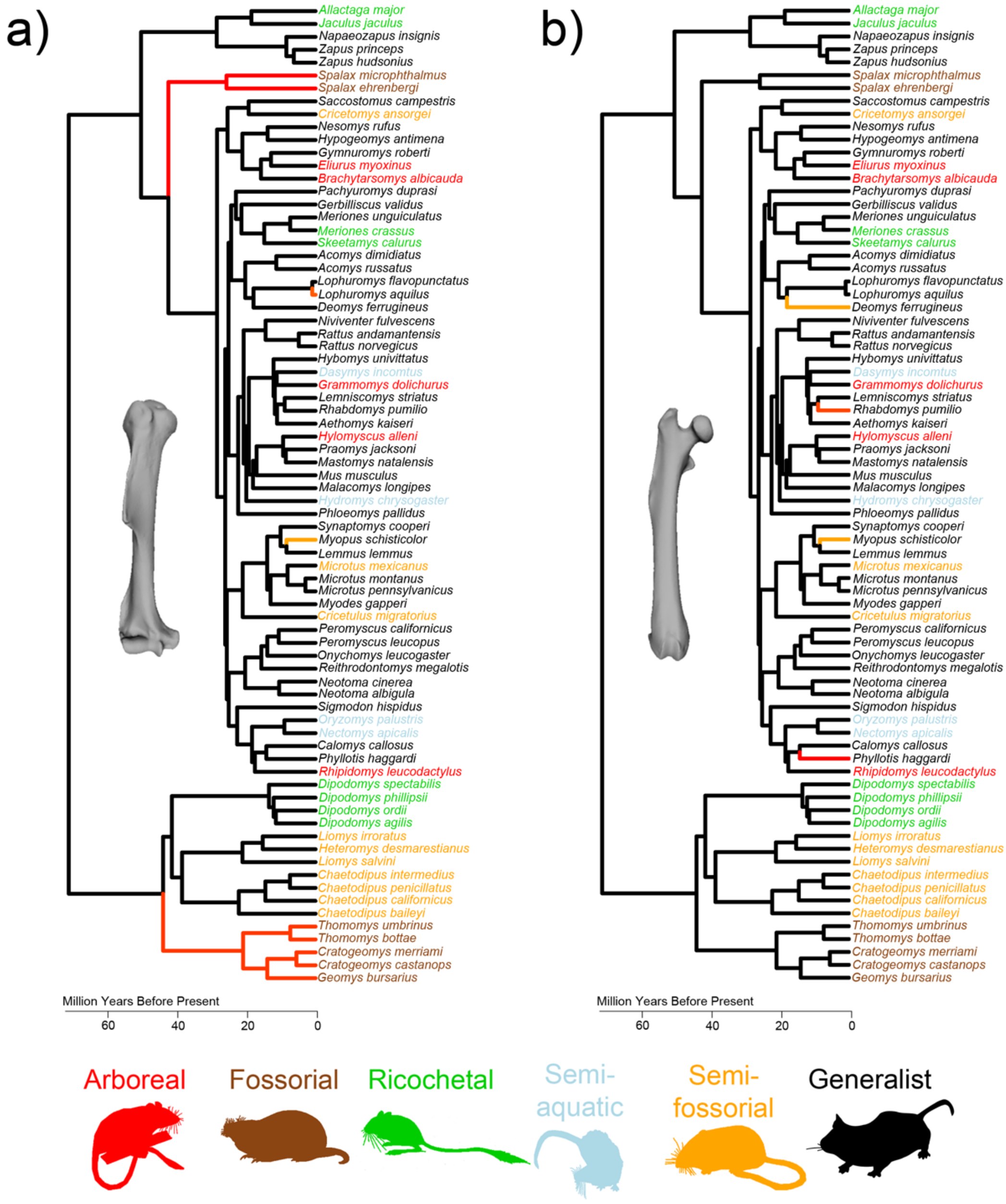

Figure S8: A MOTMOT analysis using the first 25 principal components of **(a)** humerus shape data and **(b)** femur shape data. Black branches indicate no rate shifts, red rate shifts have strong support, and orange rate shifts do not have strong support. MOTMOT sets the number of rate shifts *a priori* and four rate shifts were used in both analyses. Taxon names are colored by locomotor ecology.

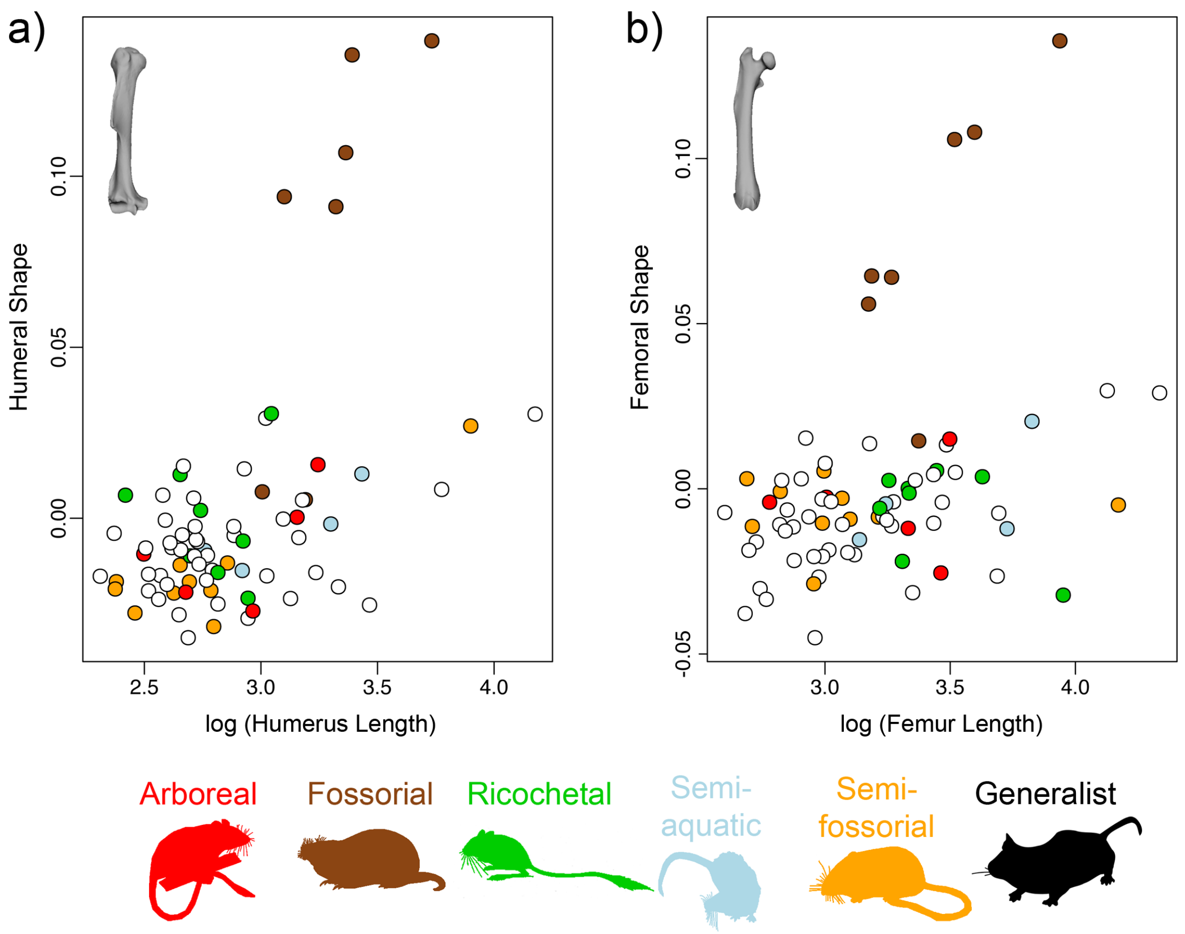

Figure S9: Allometric analysis of the common allometric component of **(a)** humeral shape and **(b)** femoral shape by log-transformed humerus and femur length respectively. Points are colored by locomotor ecology.

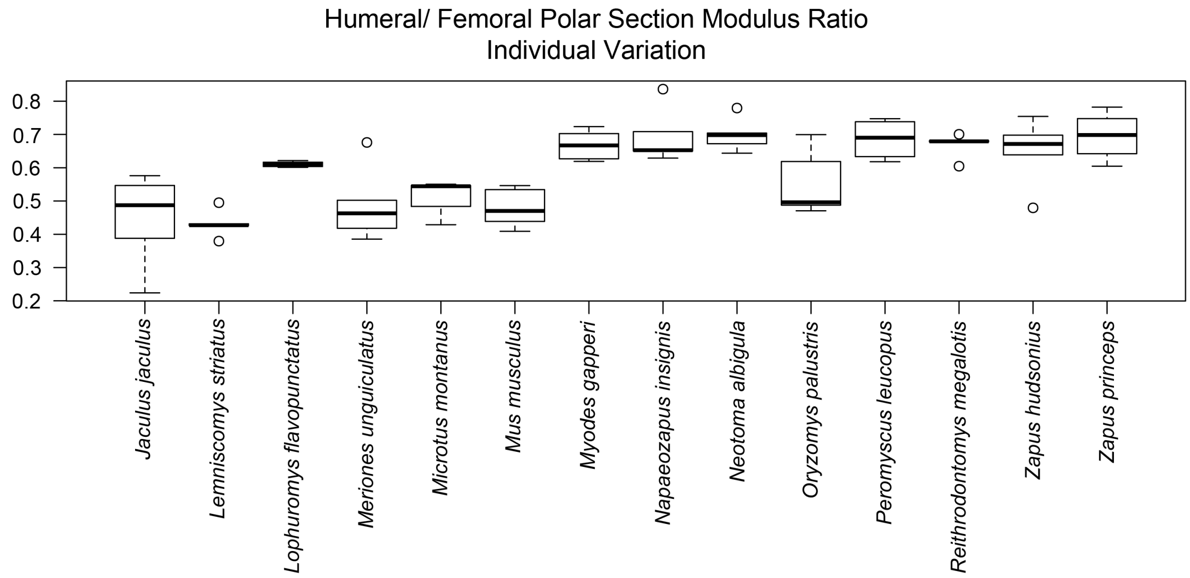

Figure S10: To assess individual variation, all taxa for which more than five individuals were measured have their range of polar section moduli shown. *Jaculus jaculus* is the only taxon represented by more than six individuals (n = 10) and further it is based on a captive sample. Generally, all taxa have a range < 0.2 suggesting that species means and taxa only represented by either one or two individuals offer an adequate representation of internal parameters.
